# On an Insidious Form of Puerperal Fever

**Published:** 1831

**Authors:** 


### XLIII.

### ON AN INSIDIOUS FORM OF PUERPERAL FEVER. By M. LEGALLOIS, Physician to the Hospice St. Michael.

IN a memoir which the author presented to the Royal Academy of Medicine, he maintained that the majority of puerperal diseases of an inflammatory nature, as phlegmasia dolens, peritonitis puerperalis, &c. were occasioned by some specific or deleterious substance mixed with the blood—and secondly, that this morbific principle was lochial discharge absorbed after it had become purulent. This etiology he based on the anatomical condition of the uterine veins—on comparative pathology, and on close examination of the various phenomena. In these ideas he was confirmed by the researches of M. Dance and various other pathologists. The following observations we shall quote in an abridged form.

The Spring of 1828 was very wet, and the Summer as dry. This constitution of the atmosphere produced immense numbers of intermittents. Scarcely a day passed without applications at the Maison Royale de Charenton. In the month of August, when the heat was very great, M. Legallois was called to a female, who had been delivered a few days previously, in an easy and natural manner. The lochia were still flowing freely, and there was no local pain of any kind. The abdomen, however, was rather more voluminous than natural, but quite void of tenderness on pressure. Every evening the female was seized with a shivering, followed by reaction, which lasted the greater part of the night. Several days were allowed thus to pass, without the exhibition of any medicine, M. L. conceiving that it was a case of the prevailing intermittent. Fearing that the paroxysms, if continued, might determine a congestion in some of the abdominal organs, our author exhibited cautiously the sulphate of quinine. The subsequent remission was prolonged beyond the usual period, and the succeeding paroxysm less severe. The quantity of quinine was increased to ten grains in the day, and ultimately to fifteen grains, without any other apparent effect than a slight amendment. But suddenly the accession became anticipated and exasperated—vomitings ensued—the epigastrium became tender, though not swelled. These symptoms continued for another day, and then a consultation was held, when it was determined that there was inflammation of the stomach, with the addition of puerperal fever. Leeches and baths were employed; but the abdomen now became distended, the vital powers were prostated, and the patient died in four or five days from the invasion of this new train of symptoms. No dissection ensued; but the Doctor very candidly blames himself for giving the quinine too long, when he ought to have watched the local symptoms. We shall give some particulars of another case, on account of the dissection.

*Case* 2. A female, 18 years of age, strong and well formed, was confined in natural labour, and safely delivered, the 9th of October, 1830. The lochia flowed during two days; on the third they became pale, and on that evening she was seized with a rigor very severe. This was succeeded by strong febrile reaction, and a remission. Next evening another rigor—and the same happened every day till the 14th October, without any symptom of local affection. Nothing but diluents were given, with an emollient lavement evening and morning. On the 15th October, the vascular action being considerable, a small quantity of blood was taken from the arm, after which the pulse rose in strength, and amounted to 120 in the minute. Still the abdomen was soft and not tender on pressure. On this day she had two rigors. 16th. She complained of great debility, and the pulse was rather weak, at 130 in the minute. For the first time pressure on the right hypogastric region occasioned deep-seated pain. The tongue was moist; and there was neither nausea nor vomiting. The rigor occurred to-day, followed by fever, and afterwards by some perspiration. In the evening all things appeared worse — the abdomen was painful—the features sunk. Another venesection, which was too late, as she died the next day.

*Dissection.* About two pints of a milky fluid were found in the cavity of the peritoneum. The peritoneum itself was neither inflamed nor injected. The uterus was about the size of two large fists, and was capable of holding a hen's egg. The place where the placenta had been attached, was rugous and pierced by several apertures, into which a quill could easily be introduced. The veins of the uterus, the muscular tissue of the organ, the ovaries, and the spermatic veins were unaltered. In short, our author was unable to trace any vestige of uterine phlebitis or uterine inflammation of any kind. He was about to give over the investigation when, on looking at the vagina, he perceived, on the internal surface of the labia, near the posterior commissure, two lacunæ, of three or four lines in diameter, filled with a white and puriform matter. On introducing a probe into one of these he found that it communicated with one of the large veins which ran from the bladder to the vagina. This vein, and the trunk from which it rose, till it merged in the hypogastric vein, viz. during an extent of three or four inches, was completely filled with the same puriform matter observed in the above-mentioned lacunæ. The veins of the opposite side presented the same phenomena, and the internal surfaces of all these vessels were found to be thickened and inflamed. The hypogastric veins contained some clots of blood mixed with a substance similar to that already mentioned. The other particulars of the dissection we pass over, as not throwing any light on the disease.—Revue Medicale.

